# An Ecological Study of the Determinants of Differences in 2009 Pandemic Influenza Mortality Rates between Countries in Europe

**DOI:** 10.1371/journal.pone.0019432

**Published:** 2011-05-11

**Authors:** Georgios Nikolopoulos, Pantelis Bagos, Theodoros Lytras, Stefanos Bonovas

**Affiliations:** 1 Hellenic Centre for Disease Control and Prevention, Athens, Greece; 2 Department of Computer Science and Biomedical Informatics, University of Central Greece, Lamia, Greece; University of Hong Kong, Hong Kong

## Abstract

**Background:**

Pandemic A (H1N1) 2009 mortality rates varied widely from one country to another. Our aim was to identify potential socioeconomic determinants of pandemic mortality and explain between-country variation.

**Methodology:**

Based on data from a total of 30 European countries, we applied random-effects Poisson regression models to study the relationship between pandemic mortality rates (May 2009 to May 2010) and a set of representative environmental, health care-associated, economic and demographic country-level parameters. The study was completed by June 2010.

**Principal Findings:**

Most regression approaches indicated a consistent, statistically significant inverse association between pandemic influenza-related mortality and per capita government expenditure on health. The findings were similar in univariable [coefficient: –0.00028, 95% Confidence Interval (CI): –0.00046, –0.00010, p = 0.002] and multivariable analyses (including all covariates, coefficient: –0.00107, 95% CI: –0.00196, –0.00018, p = 0.018). The estimate was barely insignificant when the multivariable model included only significant covariates from the univariate step (coefficient: –0.00046, 95% CI: –0.00095, 0.00003, p = 0.063).

**Conclusions:**

Our findings imply a significant inverse association between public spending on health and pandemic influenza mortality. In an attempt to interpret the estimated coefficient (–0.00028) for the per capita government expenditure on health, we observed that a rise of 100 international dollars was associated with a reduction in the pandemic influenza mortality rate by approximately 2.8%. However, further work needs to be done to unravel the mechanisms by which reduced government spending on health may have affected the 2009 pandemic influenza mortality.

## Introduction

On 11 June 2009, two months after the first human infections with a novel A (H1N1) virus of swine origin were reported from Mexico and the USA [1], the World Health Organization formally confirmed the first pandemic of influenza for 40 years [2]. To date, one year after its emergence, the pandemic A (H1N1) 2009 virus has spread across the globe having caused at least 18,000 confirmed and notified deaths [3].

In a pandemic, infection and death rates are expected to affect countries in different ways. Poor populations do endure a disproportionate burden of disease and death [4], as demonstrated by contemporary studies after the 1918 influenza pandemic [5]. In particular, the mortality rates from the 1918 pandemic were far higher in poor countries, such as India and Iran, than in Europe and North America [6], [7].

Although the 2009 influenza pandemic may be characterized as moderate in severity [2], having caused a rather small number of deaths, mortality rates were considerably higher among indigenous populations [8], [9] and varied widely from one country to another. Socioeconomic factors such as income, unemployment rates, average education level, nutritional status, comorbidities, population density and mixing rates, access to health care and quality of health system resources, as well as environmental factors may account for the observed variations in mortality rates.

The objectives of this ecological study were to identify potential, country-level, socioeconomic determinants of pandemic A (H1N1) 2009 mortality and explain between-country variation, based on data from a total of 30 countries of the European Union (EU) and the European Free Trade Area (EFTA) on which we have complete data on variables of interest.

## Methods

### Data collection

We included all confirmed and notified fatal 2009 pandemic influenza A (H1N1) cases reported from the 30 EU and EFTA countries, as of week 16, 2010 (May 2009 to May 2010). These countries operated comparable surveillance systems, with similar testing procedures for the novel A (H1N1) virus and the reporting of national data at the European level was coordinated by the European Centre for Disease Control and Prevention (ECDC). All cases of pandemic influenza met the laboratory criteria for confirmation according to the EU case definition, which included confirmation by RT-PCR, viral culture or a four-fold increase in influenza specific neutralizing antibodies (European Commission Decision of 30 April 2009; Available at: http://eurlex.europa.eu/LexUriServ/LexUriServ.do?uri=OJ:L:2009:110:0058:0059:EN:PDF).

Three representative indicators of four parameters (environmental, health care-related, economic and demographic), which might had affected pandemic A (H1N1) 2009 mortality, were selected. Unless stated differently, the data was taken from Eurostat, which is the statistical office of the European Union (http://epp.eurostat.ec.europa.eu). The study, including the retrieval of data from the publicly available electronic databases, was completed by June 2010.

### Environmental parameters

The set of environmental variables included greenhouse-gas emissions, concentration of particulate matter and geographical latitude. Exposure to gaseous pollution has been found to affect respiratory diseases such as asthma in children or chronic obstructive pulmonary disease in adults and contribute to overall mortality [10]. Moreover, gaseous pollution might increase the risk of infections and exacerbate the inflammatory effects of viral diseases in the lower respiratory tract, especially in individuals with pre-existing airway disorders. Although the underlying pathogenetic mechanisms are not fully understood, exposure to common gaseous pollutants affects the susceptibility to and the progression of infectious diseases through the impairment of local bronchial immunity, the modification of alveolar macrophages function and the epithelium damage [11]. To model the potential effect of gaseous pollution, we selected a variable providing total emissions, translated in carbon dioxide equivalents and reported as indices, with the base year  = 100 (EU-27, Euro area 15, Cyprus and Malta base year  = 1990), of four greenhouse gases (carbon dioxide, methane, nitrous oxide, sulphur hexafluoride) and two groups of gases (hydrofluorocarbons and perfluorocarbons) covered by the Protocol of Kyoto.

The second environmental indicator is a population weighted annual mean concentration of particulate matter at urban background stations in agglomerations. This variable was selected because fine particulates, i.e. particulates whose diameter is less than 10 micrometers, can reach the lungs causing inflammation and aggravating the condition of people with an underlying heart or lung disease. Long exposure to fine particulates has been associated with increased risk of worsening asthma and reduced lung function in children [12], and higher cardiopulmonary mortality [13]. Finally, in order to address the potential effects of climate factors, we also used the geographical latitude of the 30 EU and EFTA countries. The average latitude was recorded as an angular measurement in degrees, which, subsequently in the analysis, was expressed singularly with both minutes and seconds incorporated as a decimal number.

### Health care resources-related parameters

Public spending on health is a core factor in determining health outcomes [14], [15], especially for the poor [16], and, although not universally accepted as a powerful determinant of overall mortality, it might also influence, to some degree, the probability of death [17]–[19]. Therefore, the per capita government expenditure on health, expressed in international dollars and calculated using purchasing power parities (PPPs), was extracted from the World Health Information Statistical Information System (WHOSIS). PPPs can be used as currency conversion rates to express expenditures provided in national currencies into an artificial currency, thus eliminating the effect of price level variability across countries. Commonly, the PPP exchange rate refers to the number of units of a country's currency needed to purchase the same quantity of goods and services in local market, as a United States (US) dollar would buy in the US at a given point in time. The international dollar is, therefore, a hypothetical unit of currency used to translate and compare costs from one country to the other having as reference point the US dollar (http://www.who.int/choice/costs/ppp/en). To further assess the potential effect of the health care services infrastructure in each country, we also used the reported number of beds per 100,000 inhabitants and the share of the population who declared an unmet need for medical treatment or examination.

### Economic parameters

A country's income per capita and the inequality of income distribution might also account for differences in mortality rates across countries. In our analysis, the set of economic indicators contained the Gini coefficient, the Gross Domestic Product (GDP) per capita and the employment rate. The Gini coefficient is commonly used to quantify the degree of inequality in income distribution in a given society taking values between 0 and 1. It is usually multiplied by 100 to range between 0 and 100. Lower values of Gini coefficient are indicative of a more equal income distribution, with 0 corresponding to a society in which each member receives exactly the same income. Higher coefficients denote an unequal distribution with 1 indicating maximum inequality. Although the detrimental effects of unequal income distribution have been questioned by some researchers [20], Gini coefficients of national income inequality have been correlated with life expectancy and infant mortality rates [21], [22].

GDP might be the most widely used measure of the state of economy but it does not integrate all aspects that affect standard of living in a society. However, GDP per capita was selected as an indicator of an economically productive society, which provides goods and services contributing to happiness and health in the population. The association between economic development and health progress seems to follow a particular pattern. The gains in health from economic expansion are prominent in poor countries, but once a country reaches a threshold of $ 5,000–10,000 in GPD per capita, there is only a limited health benefit thereafter [23], [24]. Interestingly and counterintuitive to nature, latest ecological research has suggested that mortality fluctuates upwards during years of economic growth and downwards during recessions [24], [25]. The paradoxical improvements seen during economic recessions have been attributed to personal health-related parameters, such as the potentially increased leisure time, the decline of adverse behaviors including smoking, alcohol use or overeating, the lack of the workplace-associated stress or to the reduction in deaths occurred among the elderly from causes like motor vehicle crashes [24]. On the other hand, economic growth does not imply higher wages and individuals might work more hours, often at several jobs, to obtain an adequate income. Work intensification could adversely affect the well-being of the employees [24]. It should be noted however that in nations with larger social benefits, the health impact of business cycles is less pronounced [24]. The index of per capita GDP in Purchasing Power Standards (PPS) was expressed in relation to EU-27 average set to equal 100. If a country index was higher than 100, this country's level of GDP per head was higher than the EU average and vice versa. As explained previously with international dollar, PPS is a common artificial currency that removes variation in price levels between countries permitting meaningful comparisons.

In the analysis we also chose the employment rate as the third economic variable. Compared with other indicators, the employment rate might better reflect the consequences of economic uncertainty and insecurity faced by the population. It is postulated, based on individual studies [26], [27] that unemployment can contribute to mental health or addiction problems, to the adoption of unhealthy life styles, to loss of health insurance and, consequently, to poor disease management and ill health. However, during the years of economic boom, ecological studies have revealed an inverse relationship between job loss and mortality, especially for causes of death such as motor vehicle crashes, cardiovascular disease, influenza and pneumonia [24], [25]. On the other hand, rapid and large rises in unemployment, a common characteristic of economic turmoil, have been linked to premature deaths from intentional violence [24], [28].

### Demographic parameters

The demographic parameters in the analysis consisted of the proportion of population aged>65, the old age dependency ratio, i.e. the ratio between the total number of elderly persons of an age when they are generally economically inactive (aged 65 and over) and the number of persons of working age (from 15 to 64), and the number of women per 100 men. Studies on risk factors for death among cases infected with the pandemic A (H1N1) 2009 virus have already shown different fatality rates between the various age groups [29], [30]. Moreover, pandemic influenza in pregnancy was associated with increased hospitalization rates and severe illness, and has also been demonstrated to be a risk factor for death [29], [30]. Therefore, we modeled the aforementioned covariates in an attempt to explore the potential effect of the population composition of the countries on the variation of mortality rates.

### Statistical analysis

We used a random effects Poisson regression model to study the relationship between pandemic A (H1N1) 2009 mortality rates and a set of environmental, health care-associated, economic and demographic country-level parameters. The random effects approach was selected to account for the observed variability in the reported number of fatalities. The random effects are summarized on the basis of their estimated variances/covariances and, in this case, took the form of random intercepts for each participating country.

The following Poisson model for the number of observed deaths (μ_i_) attributable to pandemic A (H1N1) 2009 virus was specified:

log(μ_i_) = β_0_ + β_1_*Gas emissions + β_2_*Particulate matter concentration + β_3_*Average country latitude + β_4_*Hospital beds per 100,000 inhabitants + β_5_*Per capita government expenditure on health + β_6_*Percentage of people with unmet health needs + β_7_*Gini coefficient + β_8_*Gross domestic product + β_9_*Employment rate + β_10_*Percentage of people aged>65 + β_11_*Age dependency ratio + β_12_*Female to male ratio + log(Population) + u_i_ (1)

In equation 1, i stands for country (i = 1,…,30). In the standard random-effects model, u_i_ is assumed to be identically distributed such that exponentiated u_i_ is gamma with mean one and variance a, which is estimated from the data.

For model building, a stepwise backward elimination procedure was performed. Starting from the fully saturated model, we eliminated the least significant variable at each step. In the context of the multivariable analysis, we also present estimates obtained from the fully adjusted model and the model that includes significant covariates from the univariable step.

All regression estimates are presented along with their corresponding 95% confidence intervals (CI) and p-values. The tests of significance are two-sided. A probability level less than 0.05 was considered statistically significant. Stata 10 software was used for the statistical modeling and analysis (STATA, College Station, Texas, USA). The random-effects Poisson model was fitted via maximum likelihood using the xtpoisson command.

## Results

Cumulatively, 2896 fatalities, attributed to pandemic A (H1N1) 2009 strain, were reported from the 30 EU and EFTA countries. Among the studied countries, the smallest number of deaths was reported in Iceland (n = 2) and the largest in the United Kingdom (n = 474). In terms of mortality, the highest annual incidence was observed in Estonia (15.7 cases per million population) followed by Latvia (15.0) and Hungary (13.4). Table 1 shows the number of deaths in each country, the corresponding mortality rate, and the environmental, health, economic and population characteristics of each country, which might had been associated with pandemic influenza death rate.

**Table 1:** Number of fatal 2009 pandemic influenza cases, corresponding mortality rate, and the environmental, health, economic, and population characteristics of each country.

Country	Fatal cases	Population	Mortality (per million)	Gas emissions^1^	PM emissions^2^	Latitude	Hospital beds	Per capita government expenditure on health^3^	Share of the population with unmet health needs	Gini coefficient^4^	Gross Domestic Product^5^	Employment rate	Percentage of people aged >65	Age dependency ratio^6^	Female to male ratio
Austria	40	8 355 260	4.8	110.8	22.9	47.33	777.9	2 729	0.6	26	123	72.1	17.1	25.4	105.5
Belgium	19	10 750 000	1.8	92.9	26.0	50.83	660.1	2 264	0.5	28	115	62.4	17.1	25.8	104.2
Bulgaria	40	7 606 551	5.3	62.6	52.7	43	638.1	443	11.7	36	41	64.0	17.3	25.0	106.5
Cyprus	8	796 875	10.0	193.9	–	35	375.5	759	3.0	28	98	70.9	12.5	17.8	102.6
Czech Republic	102	10 467 542	9.7	72.5	29.8	49.75	727.3	1 309	0.3	25	80	66.6	14.6	20.5	104.2
Denmark	33	5 511 451	6.0	92.6	21.4	56	340.8	2 812	0.0	25	117	78.1	15.6	23.6	101.9
Estonia	21	1 340 415	15.7	49.6	11.1	59	557.3	734	0.9	31	62	69.8	17.2	25.3	117.2
Finland	44	5 326 314	8.3	99.7	14.3	64	673.6	1 940	0.5	26	110	71.1	16.5	24.8	104.1
France	344	64 350 759	5.3	93.6	24.1	46	700.3	2 833	1.6	28	107	64.9	16.3	25.1	106.7
Germany	254	82 002 356	3.1	77.8	21.1	51	829.1	2 548	1.6	30	116	70.7	19.9	30.0	104.1
Greece	141	11 260 402	12.5	122.8	36.8	39	473.8	1 317	4.3	33	95	61.9	18.6	27.8	101.9
Hungary	134	10 030 975	13.4	75.1	27.1	47	713.3	978	2.5	25	63	56.7	16.2	23.5	110.6
Iceland	2	319 368	6.3	142.9	11.5	65	–	2 758	1.2	27	120	83.6	11.5	17.1	96.1
Ireland	25	4 450 030	5.6	123.0	13.7	53	519.9	2 413	1.2	30	131	67.6	10.9	15.9	100.3
Italy	244	60 045 068	4.1	104.7	34.3	42.83	386.3	2 022	3.9	31	102	58.7	20.0	30.4	105.9
Latvia	34	2 261 294	15.0	44.4	23.8	57	744.5	615	6.9	38	49	68.6	17.2	24.9	116.9
Lithuania	23	3 349 872	6.9	48.9	17.4	56	816.2	728	1.8	34	53	64.3	15.8	23.0	114.8
Luxembourg	3	493 500	6.1	95.2	–	49.75	571.4	5 233	0.5	28	268	63.4	14.0	20.6	101.9
Malta	5	413 609	12.1	144.2	29.3	35.83	737.3	1 419	0.5	27	78	55.3	13.5	19.3	101.0
Netherlands	62	16 485 787	3.8	97.6	25.2	52.5	481.5	2 768	0.0	28	130	77.2	14.7	21.8	102.2
Norway	29	4 799 252	6.0	108.0	18.9	62	382.3	3 780	0.2	25	177	78.0	14.6	22.1	100.8
Poland	180	38 135 876	4.7	87.3	33.4	52	642.5	636	2.7	32	56	59.2	13.5	18.9	107.0
Portugal	122	10 627 250	11.5	132.2	24.3	39.5	365.1	1 494	0.9	36	78	68.2	15.7	23.4	106.6
Romania	122	21 498 616	5.7	60.3	41.1	46	641.1	433	10.0	36	42	59.0	14.9	21.3	105.2
Slovakia	56	5 412 254	10.3	66.1	25.0	48.67	674.9	913	0.5	24	72	62.3	12.0	16.6	105.9
Slovenia	19	2 032 362	9.3	115.2	29.9	46	473.2	1 507	0.1	23	86	68.6	16.3	23.3	103.8
Spain	271	45 828 172	5.9	142.3	27.7	40	330.2	1 732	0.1	31	104	64.3	16.6	24.1	102.5
Sweden	27	9 256 347	2.9	88.3	17.6	62	287.7	2 533	0.6	24	120	74.3	17.5	26.7	101.2
Switzerland	18	7 701 856	2.3	100.5	–	47	539.3	2 598	–	–	144	79.5	16.4	24.1	103.7
United Kingdom	474	61 595 961	7.7	81.4	20.4	54	341.8	2 434	0.1	34	116	71.5	16.1	24.3	103.7

Statistics were obtained from EUROSTAT.

Data on per capita government expenditure on health was derived from the World Health Information Statistical Information System (WHOSIS).

1 Total emissions in CO_2_ equivalents presented as indices, with the base year = 100.

2 Population weighted annual mean concentration of particulate matter at urban background stations in agglomerations.

3 Expressed in international dollars, which are calculated using purchasing power parities (PPP) (PPP - rates of currency conversion constructed to account for differences in price level between countries).

4 Index of inequality of income distribution ranging from 0 (complete equality) to 100 (complete inequality).

5 The index is calculated from Purchasing Power Standards (PPS) figures and expressed with respect to European Union-27 = 100.

6 The ratio between the total number of persons of aged 65 and over, and the number of persons aged between 15 and 64.

**Figure 1:**
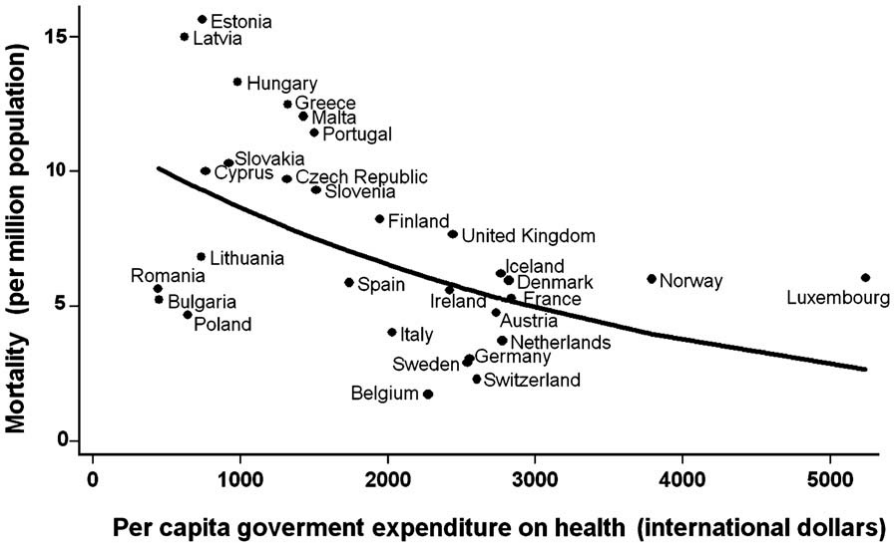
Relation between per capita government expenditure on health and pandemic A (H1N1) 2009 mortality in 30 European countries. The superimposed line is obtained by a random-effects Poisson regression model.

Table 2 summarizes the results of the regression modeling, which shows a consistent, statistically significant inverse association between pandemic influenza-related mortality and per capita government expenditure on health. More specifically, in the univariable analyses, the random-effects Poisson regression approach provided a negative coefficient of –0.00028 for the per capita government expenditure on health (95% CI: –0.00046, –0.00010, p-value = 0.002). Figure 1 depicts graphically the association between pandemic mortality rate and public spending on health. The GDP, on per capita basis, was also inversely related to death rate (coefficient: –0.00631, 95% CI: –0.01112, –0.00151, p-value = 0.010) in univariable analyses. Finally, the female-to-male ratio was the third index, which was associated with mortality. The estimated coefficient describing its impact was 0.04798 (95% CI: 0.00960, 0.08636, p-value = 0.014).

**Table 2:** Association of covariates with pandemic influenza mortality rates.

Covariates^1^	Univariable analysis^2^	95% CI	p-value	Multivariable analysis including all covariates^2^	95% CI	p-value	Multivariable analysis with significant covariates from univariable analysis^2^	95% CI	p-value
**Environmental parameters**									
Greenhouse gas emissions^3^ (2008)	–0.00111	–0.00680, 0.00458	0.702	0.00012	–0.01090, 0.01115	0.983			
Concentration of particulate matter^4^ (2007–2008)	–0.00410	–0.02596, 0.01776	0.713	–0.02156	–0.07229, 0.02918	0.405			
Geographical latitude	–0.00790	–0.03204, 0.01624	0.521	–0.05095	–0.09792, –0.00398	0.034			
**Health care resources–related parameters**									
Hospital beds per 100,000 inhabitants (latest available)	0.00029	–0.00092, 0.00150	0.639	–0.00038	–0.00159, 0.00082	0.532			
Per capita government expenditure on health^5^ (2006)	–0.00028	–0.00046, –0.00010	0.002	–0.00107	–0.00196, –0.00018	0.018	–0.00046	–0.00095, 0.00003	0.063
Unmet need for medical examination/treatment (2008)	0.01124	–0.05621, 0.07868	0.744	0.03591	–0.08143, 0.15325	0.549			
**Economic parameters**									
Gini coefficient^6^ (2008)	0.01428	–0.02666, 0.05522	0.494	–0.04507	–0.09437, 0.00422	0.073			
Gross domestic product^7^ per capita (2009)	–0.00631	–0.01112, –0.00151	0.010	0.01732	–0.00461, 0.03925	0.122	0.00756	–0.00591, 0.02102	0.271
Employment rate (2008)	–0.02227	–0.05172, 0.00718	0.138	0.04590	0.00011, 0.09170	0.049			
**Demographic parameters**									
Proportion of population aged 65 and over (2008)	–0.02897	–0.12256, 0.06462	0.544	–0.49838	–1.59463, 0.59787	0.373			
Old age dependency ratio^8^ (2008)	–0.02676	–0.08329, 0.02978	0.354	0.27997	–0.39016, 0.95010	0.413			
Women per 100 men (2008)	0.04798	0.00960, 0.08636	0.014	0.06468	–0.00516, 0.13452	0.069	0.03154	–0.01399, 0.07706	0.175

1 Statistics were obtained from EUROSTAT. Data on per capita government expenditure on health was derived from the World Health Information Statistical Information System (WHOSIS).

2 Random-effects poisson regression model.

3 Total emissions in CO_2_ equivalents presented as indices, with the base year = 100.

4 Population weighted annual mean concentration of particulate matter at urban background stations in agglomerations.

5 Expressed in international dollars, which are calculated using purchasing power parities (PPP) (PPP - rates of currency conversion constructed to account for differences in price level between countries).

6 Index of inequality of income distribution ranging from 0 (complete equality) to 100 (complete inequality).

7 The index is calculated from Purchasing Power Standards (PPS) figures and expressed with respect to European Union-27 = 100.

8 The ratio between the total number of persons of aged 65 and over, and the number of persons aged between 15 and 64.

Incorporating the aforementioned covariates in the same model, we noted that the per capita government expenditure on health retained its effect. The multivariable model including the three significant variables derived from univariable analyses, arrived at a coefficient of –0.00046 for public spending on health, which was marginally insignificant at the 0.05 level (95% CI: –0.00095, 0.00003, p-value = 0.063). The effect of government expenditure on health per capita was significant after adjusting for all covariates considered in the current analysis (coefficient: –0.00107, 95% CI: –0.00196, –0.00018, p-value = 0.018). In the fully adjusted model, another two variables were statistically significant: the geographical latitude (coefficient: –0.05095, 95% CI: –0.09792, –0.00398, p-value = 0.034) and the employment rate (coefficient: 0.04590, 95% CI: 0.00011, 0.09170, p-value = 0.049).

Lastly, the stepwise backward elimination procedure, which was applied to estimate the best model fitted to the data, resulted in having only one significant predictor in the model, the government expenditure on health per capita. It should be noted that, in all cases, the random-effects estimators were significantly different from the conventional Poisson estimators.

Interpreting the estimated coefficient (–0.00028) for the per capita government spending on health, we observed that a rise of 100 international dollars was associated with a reduction in the pandemic influenza mortality rate by approximately 2.8%.

## Discussion

Though the 2009 influenza pandemic was considerably less lethal than originally expected, having caused a rather small number of deaths, mortality rates varied widely from one country to another. In this ecological study, we attempted to assess the potential country-level determinants of pandemic mortality and explain the between-country variation assuming reasonably that surveillance systems and reporting of fatalities were comparable among the 30 European countries. Our findings imply a significant inverse association between per capita government expenditure on health and pandemic influenza mortality. The significant association of mortality with other covariates such as the employment rate or the geographical latitude was not consistently observed in all models applied and valid conclusions regarding their potential effects, based solely on statistical grounds, cannot be drawn.

A previous study [31] has indicated a strong negative association between per-head income and mortality in the 1918 influenza pandemic. Interestingly, in the current study regarding the 2009 pandemic the GDP effect did not retain its significance throughout the analysis, while it appears that this time public spending on health, which is a social indicator of the degree of investment in the human capital, has a core role to explain variations in mortality. The multivariable-adjusted estimates obtained from regression analyses led to similar findings, a fact that reinforces our confidence in the validity of the observed association.

There is extensive evidence that reduced public expenditure allocations to the health sector have adverse consequences for the health of populations, which substantiates the ecological findings of this analysis. More specifically, previous research has suggested that, although economic progress, especially in low-income countries, impacts health outcomes such as under-five mortality, government spending on health is an equally important contributor [15], [18]. Furthermore, less public expenditure on health appeared to be a key hazard to infant survival [15], [32], a finding that has also been observed in the European setting [17]. Even though not closely related to the effects of government expenditure on health, a recent study explored the potential association between levels of social spending and age standardized all cause mortality in 15 European countries and produced extremely significant findings [33]. The analysis showed that 100$ increase in social welfare spending corresponded to 1.19% reduction in mortality. More interestingly, although GDP was also correlated to mortality a comparable increase in social spending produced a greater drop in death rate than a rise in GDP of similar magnitude and the estimated effect of GDP was almost cut by two thirds when it was adjusted for social spending in the constructed models.

Absolute wealth or economic progress is essential for the well-being of the population but does not lead to improved health per se. It seems that the appropriate expansion of public health services and their use in a socially productive way determine the health benefits of economic growth [24]. At the present time, many European countries, being hit hard by the global economic turmoil, are far from economic expansion and face an unwanted recession phase. Governments are pressed to endorse economic programs of macroeconomic stability balancing their limited budget and raising productivity, which, finally, even though not directly recommended by the inventors of these programs, constrain country policies and, subsequently, public health spending. Some analysts argue that recession might lead to health gains. Reduced investments in health, however, have been associated with worsened health outcomes [34]. Moreover, the rapidity of economic change might itself negatively affect the health of the population [35]. Therefore, based on the results of the current study and the aforementioned evidence, national governments, in order to buffer the effects of economic shifts, need to safeguard budgetary allocations to the health sector [24] taking also into account the emerging evidence that expenditure allocations in favor of health, contrary to what might have been expected, not only secure human lives but can also boost economic growth while reducing poverty [36].

Nevertheless, our study has at least three limitations. First, as with all cross-country analyses, the potential exists for spurious statistical correlations produced by unknown sources of confounding [37]. Ecological fallacies are also present if inferences about the nature of individuals are drawn based only on aggregate statistics. In other words, associations observed at the country-level might not apply at the individual members. Therefore, our findings should be interpreted with caution and further verification is needed. Second, government expenditure on health may not be the only explanation for the observed differences in pandemic mortality, but this finding offers a partial account of the ultimate correspondents of between-country variation. Third, although the analysis was restricted to European countries with, probably, comparable surveillance systems and quite similar procedures of data reporting, differences may still exist and lead to various biases. Usually, however, countries with lower government spending on health operate surveillance systems that suffer higher rates of underreporting. If such bias exists for surveillance of pandemic influenza across Europe, it would imply that the strength of the inverse association between the per capita government expenditure on health and pandemic A (H1N1) mortality, which was found in this study, might have been conservatively underestimated.

The best guides we will have for the effects on mortality of a future influenza pandemic are the studies of the previous epidemics. Therefore, in this ecological analysis, our attention was turned to the role of various country-level covariates in the pandemic A (H1N1) 2009 death rate. It can be concluded that there is a consistent and statistically significant association between per capita government expenditure on health and pandemic A (H1N1) 2009 mortality. However, this particular association is neither definite nor thoroughly clear. This analysis should be viewed within its limitations, at least, as hypothesis generating. Further work needs to be done, on individual patient databases, to unravel the mechanisms by which reduced government spending on health may have affected the 2009 pandemic influenza mortality. These may include limited access to medical care services, low quality of health system resources, inadequate numbers of health workers, underfunded influenza pandemic preparedness and ineffective public health interventions. These have long been fundamental concerns for public health, and new efforts have to be made to push them up in the global health policy agenda.
